# Intraperitoneal extension of the peritoneal dialysis catheter—a new technique for catheter implantation in patients with obesity

**DOI:** 10.1007/s40620-021-01077-z

**Published:** 2021-07-08

**Authors:** Michael Sayer, Christian Thiel, Martin Schenk, Alfred Königsrainer, Nils Heyne, Andreas L. Birkenfeld, Ferruh Artunc, Karolin Thiel

**Affiliations:** 1 Department of Internal Medicine, Division of Endocrinology, Diabetology and Nephrology, Tübingen University Hospital, Tübingen, Germany; 2 Department of General, Visceral and Transplant Surgery, Tübingen University Hospital, Hoppe-Seyler-Strasse 3, 72076 Tübingen, Germany; 3 Institute of Diabetes Research and Metabolic Diseases (IDM) of the Helmholtz Center Munich At Tübingen University, Tübingen, Germany; 4 German Center for Diabetes Research (DZD) at Tübingen University, Tübingen, Germany

**Keywords:** Extended PD catheter, Implantation, Obesity, Peritoneal dialysis catheter extended PD catheter, Implantation, Obesity, Peritoneal dialysis catheter

## Abstract

**Background:**

In patients with obesity and end-stage kidney disease, implantation of the peritoneal dialysis (PD) catheter may be complicated by increased abdominal circumference or skin folds. Relocation of the implantation site to the upper abdomen could solve this problem. However, this would require an extended catheter.

**Methods:**

We developed an extended PD catheter based on a swan neck Missouri PD catheter with the help of two adaptors and a straight intraperitoneal extension segment. The extended catheter was assembled intraoperatively, and its length was adjusted individually to ensure correct positioning. After the operation, PD was commenced and handled as usual.

**Results:**

In the period from 2011 to 2021, we implanted 31 extended PD catheters in 29 patients (38% men) with end-stage renal failure and obesity. Median age was 53 (range 28–77) years and body mass index was 35.5 (range 26.4–46.9) kg/m^2^. The postoperative course was unremarkable except for seroma formation in one patient and dialysate leakage in another. Continuous ambulatory peritoneal dialysis (CAPD) was initiated in 20 and APD in 9 patients. The achieved median Kt/V was 2.10 (range 1.50–3.10). During the follow-up period lasting up to 51 months, there was one case of intraperitoneal catheter disconnection due to an avoidable handling error. The peritonitis rate was 1:40 months. The 1- and 2-year catheter survival was 92% and 67%, respectively, and paralleled patient survival.

**Conclusions:**

When using a PD catheter with an intraperitoneal extension, PD catheter implantation can be relocated to the upper abdomen in patients with obesity, thus providing optimal position and easy surgical access.

## Introduction

Obesity has continued to rise worldwide over the past years [1] and patients with end-stage kidney disease are disproportionately often affected by obesity [2]. Compared to hemodialysis (HD), peritoneal dialysis (PD) is rarely offered as renal replacement therapy in patients with obesity [3] for reasons such as higher infection rates, early technique failure and inadequate solute clearance. However, other authors found similar dysfunction-free PD catheter survival and similar complication rates in patients with obesity [4]. Given these encouraging outcome data, obesity can no longer be considered a contraindication to PD [3].

In patients with obesity, anatomical structural conditions with increased waist circumference are challenging for PD catheter insertion. Twardowski et al. developed a presternal swan neck catheter [5] and Crabtree relocated the exit of the extended catheter to the upper abdomen for improved view and accessibility [6]. Compared to standard catheters, extended catheters in patients with obesity had longer catheter survival times and lower exit site infection rates [7]. However, these extended catheters are placed with a long subcutaneous tunneled segment that entails the risk of kinking or movement in the large subcutaneous fat layer. In addition, the technique requires two access paths, one in the chest or upper abdomen and one in the lower abdomen.

We hypothesized that a new insertion technique that relocates the PD catheter insertion to the upper abdomen could simplify the operative procedure and minimize surgical trauma. To ensure proper location of the catheter tip in the Douglas space, we extended the PD catheter by adding an intraperitoneal catheter segment. The aim of this technical note is to report the feasibility of our approach and our experiences with intraperitoneally extended catheters regarding perioperative complications, dysfunction and catheter survival.

## Materials and methods

### Study cohort

The retrospective study cohort consisted of 29 patients with obesity and end-stage kidney disease, who received an intraperitoneally extended PD catheter at Tübingen University Hospital, Germany, between 2011 and 2021. All patients had previously opted for PD as renal replacement therapy by shared decision-making with the treating nephrologist. On referral, patient selection for implantation of an extended PD catheter was based on waist circumference, or the presence of skin folds, or excess skin that otherwise would not have allowed a conventional PD catheter to be implanted at the site near the umbilicus. Siting was done in accordance with the ISPD recommendations on the correct positioning of the catheter [8].

### Preparation of the extended peritoneal dialysis catheter

**Fig. 1:**
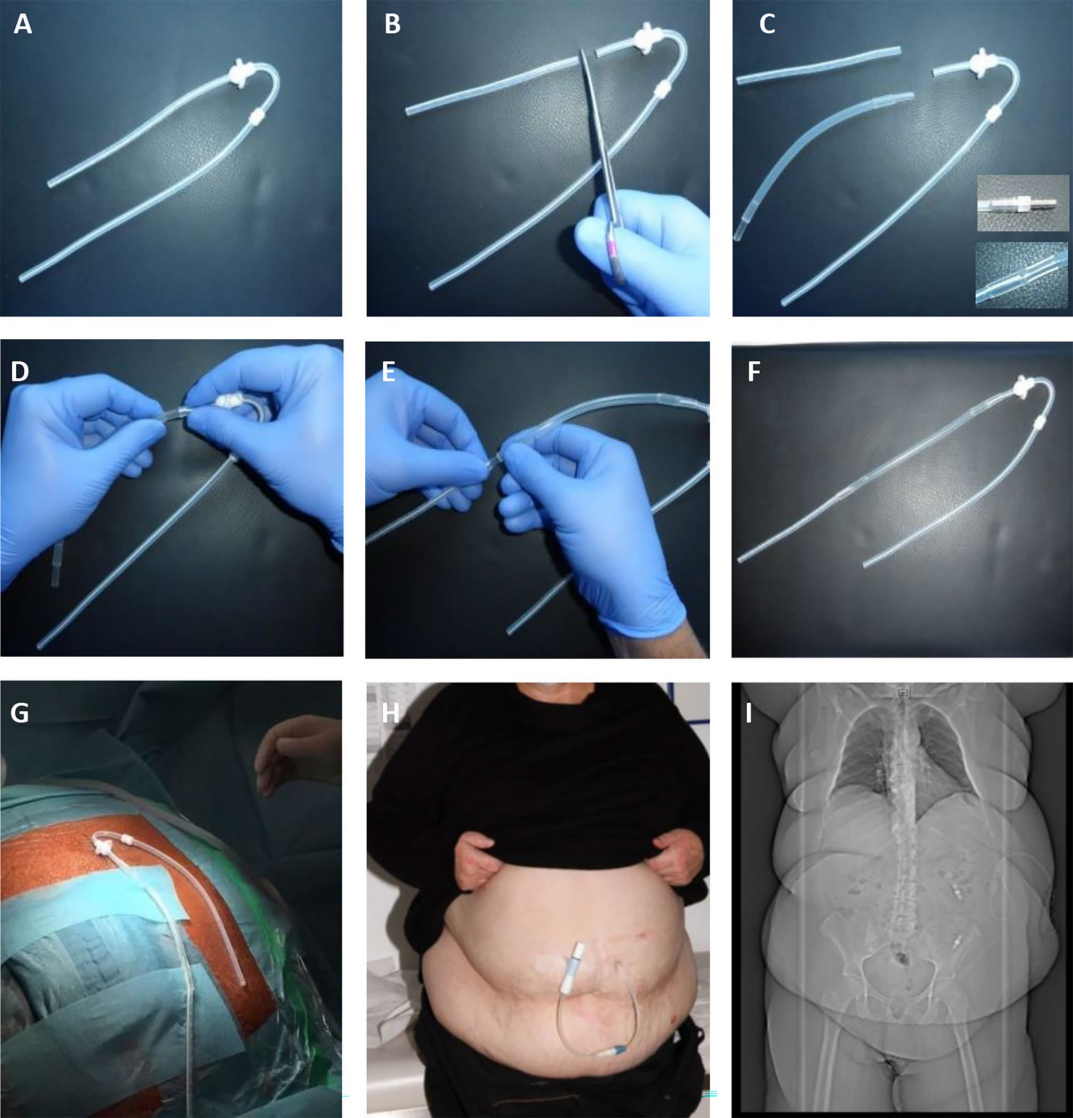
Assembly of an extended swan neck PD catheter and position of the PD catheter in a representative patient with obesity (BMI 47 kg/m^2^). After cutting off the intraperitoneal segment of the swan neck PD catheter (**A**, **B**), two titan (Covidien 8888–415,612) or silicon adaptors (Oriplast, Neunkirchen, Germany REF 260.480) were mounted on a flexible silicon rubber tube that was adjusted to the patient’s figure and connected to the proximal end (**C**–**E**). Finally, the extended catheter was completed using the intraperitoneal segment that had previously been cut off (**F**). These steps were performed in the OR under sterile conditions. Intraoperatively, the implantation position in the upper abdomen was adjusted according to the patient’s figure (**H**). Position of the catheter after implantation with the patient standing (**H**) or lying for CT (**I**), which was performed for a different reason. Note the two titan adaptors in the left upper and lower abdomen

All operations were performed by high-volume visceral surgeons. Dual cuffed PD catheters with a deep disk-and-ball cuff and either a straight or curled intraperitoneal segment (swan neck Missouri or Oreopoulus-Zellermann, Covidien, Dublin, Ireland) were used to prepare an extended PD catheter as stepwise shown in Fig. 1A–H.

### Surgical technique

Standardized preoperative mapping in a standing, sitting and lying position to select the most suitable catheter position was performed by the PD care team one day before the operation. After assembling the extended catheter, the implantation site was verified in the operating room (Fig. 1H).

Standard prophylactic antibiotic treatment consisted of 2 g i.v. cephazolin immediately before, and 6 and 24 h after implantation. Catheter implantation was performed under general anesthesia using a modified version of the implantation technique described by Twardowski et al. [5]. In contrast to Twardowski [5], a vertical paramedian incision of 3–4 cm in the upper abdomen was performed as operational access and the exit site was chosen approximately 3 cm lateral to this incision. The extended catheter was placed deep in the peritoneal cavity using a long stiffening stiletto (Covidien 57 cm 8888–415 661) which is 2–3 cm shorter than the extended catheter and avoids injury of the intestine during positioning.

### Data collection and statistics

After patient discharge, follow-up data were collected from patients and treating nephrologists by phone interview. Data analysis was approved by the Tübingen University Ethics Committee (194/2018BO). Values are given as medians with the complete range or the 95% confidence interval, as indicated. Patient and catheter survival curves were calculated with the Kaplan–Meier method and tested for significance using a log-rank test. A p value < 0.05 was considered significant. Statistical analysis was done using MedCalc Statistical Software, version 19.1.3 (MedCalc Software bv, Ostend, Belgium; https://www.medcalc.org).

## Results

The retrospective study cohort comprised 29 patients with obesity receiving 31 intraperitoneally extended PD catheters at Tübingen University Hospital between 2011 and 2021. One patient received three extended catheters. Median body mass index (BMI) was 35.5 (range 26.4–46.9) kg/m^2^. Further patient characteristics are shown in Table 1. No intraoperative complications occurred (Table 2). Postoperatively, one patient developed a seroma of the paramedian incision and one patient developed dialysate leakage. Figure 1 depicts the assembly of an extended PD catheter (A–H) and the position after implantation in a representative patient as studied in an abdominal X-ray and CT scan (I, J).

**Table 1:** Patient characteristics at the time of PD catheter implantation

N	29
Males/females	11/18
Age, years	53 (28–77)
Renal disease	Diabetic nephropathy (n = 7)
	Polycystic kidney disease (n = 7)
	Nephrosclerosis (n = 7)
	IgA-glomerulonephritis (n = 4)
	Allograft failure (n = 2)
	Cardiorenal syndrome (n = 1)
	Unknown (n = 1)
Prior abdominal surgery	14
Height, (cm)	170 (160–189)
Weight, (kg)	104 (70–140)
Body mass index, (kg/m^2^)	35.0 (26.4–46.9)
Plasma creatinine, (mg/dL)	6.3 (3.6–9.9)
Estimated GFR, (mL/min/1.73 m^2^)	8.8 (4.0–15.4)
Plasma urea, (mg/dL)	152 (51–245)
Plasma Na^+^, (mM)	140 (132–143)
Plasma K^+^, (mM)	4.8 (3.4–6.2)
Hemoglobin, (g/dL)	10.4 (8.0–15.2)

Values are given as median with range

**Table 2:** Outcome data following implantation of an extended PD catheter in patients with obesity

Patients, n	29
Patients with a single catheter implantation, n	28
Patients with three catheters, n	1
Extended catheters, n	31
Median operative time, min	38 (24 – 66)
Intraoperative complications, n	0
Early complications < 30 days postop, n	2
Seroma	1
Dialysate leakage	1
Late complications > 30 days postop, n patients	16
Intraperitoneal disconnection	1
Exit-site infection	4
Peritonitis	15
Hernia	1
Total follow-up, months	597
Median follow-up, months	25 (2–51)
Removal of the extended PD catheter, n catheters	15/31
Due to technical PD failure, n catheters	5/31
Due to peritonitis, n catheters	5/31
Due to catheter dysfunction, n catheters	2/31
Due to patient’s decision, n patient	1/29
Due to recovery of renal function, n patient	1/29
Due to transplantation, n patient	1/29
Death with a functioning catheter, n patient	8/29
Continued on PD, n patient	9/29

Median values with range

Twenty of the patients were treated with continuous ambulatory peritoneal dialysis (CAPD) and n = 9 with APD including n = 3 patients who were switched from an initial treatment with CAPD. Median daily dialysate volume was 6.0 (range 4.5–9.5) L in CAPD and 11.3 (range 9.4–16.0) L in APD. The achieved median Kt/V after 3 months was 2.0 (range 1.50–3.10) for all patients. During the follow-up period of maximum 51 (median 25) months, no patient complained of abdominal discomfort. Overall, there were no flow problems during either CAPD or APD. Four patients developed an exit-site infection (Table 2). There were 15 episodes of peritonitis in 10 patients during 597 treatment months, which corresponds to a peritonitis rate of 1:40 months.

During follow-up 15 (48%) out of 31 extended catheters were removed and the patients were switched to hemodialysis (HD) (n = 11) or underwent renal transplantation (n = 1, Table 2). Five (16%) catheters were removed because of technical PD failure (ultrafiltration failure and/or inadequate dialysis adequacy) and five (16%) due to peritonitis. Two catheters were removed because of dysfunction, one catheter due to recovery of renal function and one catheter due to the patient’s decision to terminate PD because of excessive weight gain (Table 2). There was one case of intraperitoneal disconnection 24 months after implantation that was caused by forced manual flushing of the blocked catheter with a syringe during hospitalization for endocarditis. Subsequently, this patient was switched to hemodialysis. During follow-up, eight patients (28%) died with a functioning PD catheter, while nine (31%) continued on PD.

**Fig. 2:**
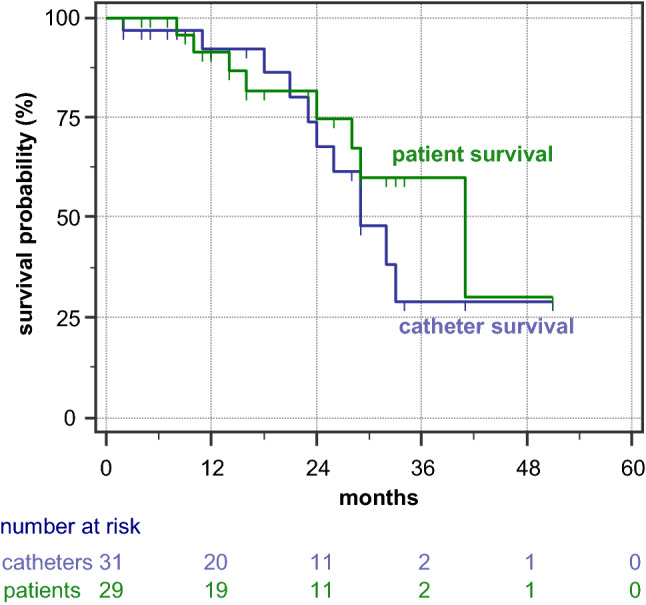
Kaplan–Meier curves for patient survival and PD catheter survival. PD catheter survival was defined as the presence of the originally implanted extended catheter and continuation of PD (n = 9). Events were catheter removal due to peritonitis (n = 5) or insufficient dialysis adequacy, leading to mandatory switch to HD (n = 5). Death with a functioning catheter (n = 8), switch to HD on patient’s decision (n = 2), recovery of renal function (n = 1) and kidney transplantation (n = 1) were considered censoring events (marked by blue ticks)

Kaplan–Meier curves for patient and catheter survival are shown in Fig. 2. Patient survival at one and two years was 92 and 71%, respectively. The 1-year and 2-year death-censored catheter survival was 92 and 67%, respectively. Median catheter survival was 29 months (95% confidence interval 23;33). Female patients (n = 17) had significantly longer median catheter survival (40 months) than did male patients (n = 12, 23 months, p = 0.0041 by log-rank).

## Discussion

Our study demonstrates the feasibility of implanting a PD catheter with an intraperitoneal extension in patients with obesity. The implantation approach was associated with a very low perioperative complication rate and a very high 1-year and 2-year catheter survival rate while achieving excellent PD quality. Our approach has proven to be simple and safe in patients with obesity, who otherwise could not have been given a PD catheter using the conventional approach, at least at our center.

Similar to Crabtree [6], we used the upper abdomen site because the subcutaneous fat layer there is reduced compared to the lower abdomen and because the skin of the upper abdomen has fewer skin folds (Fig. 1H). Instead of a long tunnel, we entered the abdomen at the same place in the upper abdomen and extended the intraperitoneal catheter segment using a custom-made approach. The main advantage of this access is that it minimizes surgical trauma with only one incision and prevents surgical complications. In the present study we observed only one seroma and one dialysate leakage. Another advantage of our insertion technique is the applicability to any catheter type and the individual adaptability of the catheter length for each patient regardless of sex. Each patient underwent standardized preoperative mapping to find the most suitable catheter position. Additionally, the intraperitoneal segment was immediately individually adjusted in the operating room. Noteworthy, there was no primary PD failure among our patients.

Ninety-two percentage of the extended catheters implanted in our study survived 1 year, and at 2 years catheter survival was 67%. Self-reported satisfaction with the catheter was good and an exit site in the upper abdomen was easily manageable for patients.

The present study is limited by its small number of patients and its single-center setting. The catheter was custom-made and not standardized. Intraperitoneal disconnection occurred in one case and was related to a handling error by forced manual flushing. Although catheter disconnection is serious and must be corrected by another surgical intervention, avoidance of manual flushing will prevent catheter disconnection in future. In the remaining patients there were no cases of spontaneous disconnection as this is almost impossible due to the tight connection of the catheter segments by the adaptors. As a strength, this study demonstrates the feasibility of this approach in highly selected patients with obesity, resulting in a very low complication rate, even in an urgent-start setting [9].

In conclusion, we developed a new PD insertion technique with an intraperitoneally extended catheter to simplify the operative procedure and minimize the surgical trauma in patients with obesity. This surgical method is associated with a very low perioperative complication rate and a very high 1-year and 2-year catheter survival rate.

## Data Availability

The data underlying this article will be shared on reasonable request to the corresponding author. The results presented in this paper have not been published previously in whole or part, except in abstract form.
